# A review of COVID-19 transmission dynamics and clinical outcomes on cruise ships worldwide, January to October 2020

**DOI:** 10.2807/1560-7917.ES.2022.27.1.2002113

**Published:** 2022-01-06

**Authors:** Kathryn S Willebrand, Lauren Pischel, Amyn A Malik, Samuel M Jenness, Saad B Omer

**Affiliations:** 1 Yale Institute of Global Health, New Haven, Connecticut, United States; 2 Yale School of Public Health, New Haven, Connecticut, United States; 3 Yale School of Medicine, Section of Infectious Diseases, New Haven, Connecticut, United States; 4 Emory University Rollins School of Public Health, Atlanta, Georgia, United States; 5 Yale School of Nursing, Orange, Connecticut, United States

**Keywords:** SARS-CoV-2, COVID-19, cruise ship, cruise ship outbreak, epidemiology

## Abstract

**Background:**

Cruise ships provide an ideal setting for transmission of SARS-CoV-2, given the socially dense exposure environment.

**Aim:**

To provide a comprehensive review of COVID-19 outbreaks on cruise ships.

**Methods:**

PubMed was searched for COVID-19 cases associated with cruise ships between January and October 2020. A list of cruise ships with COVID-19 was cross-referenced with the United States Centers for Disease Control and Prevention’s list of cruise ships associated with a COVID-19 case within 14 days of disembarkation. News articles were also searched for epidemiological information. Narratives of COVID-19 outbreaks on ships with over 100 cases are presented.

**Results:**

Seventy-nine ships and 104 unique voyages were associated with COVID-19 cases before 1 October 2020. Nineteen ships had more than one voyage with a case of COVID-19. The median number of cases per ship was three (interquartile range (IQR): 1–17.8), with two notable outliers: the Diamond Princess and the Ruby Princess, which had 712 and 907 cases, respectively. The median attack rate for COVID-19 was 0.2% (IQR: 0.03–1.5), although this distribution was right-skewed with a mean attack rate of 3.7%; 25.9% (27/104) of voyages had at least one COVID-19-associated death. Outbreaks involving only crew occurred later than outbreaks involving guests and crew.

**Conclusions:**

In the absence of mitigation measures, COVID-19 can spread easily on cruise ships in a susceptible population because of the confined space and high-density contact networks. This environment can create superspreader events and facilitate international spread.

## Introduction

Transmission of severe acute respiratory syndrome coronavirus 2 (SARS-CoV-2) is facilitated by prolonged contact and close proximity to an infectious individual in poorly ventilated settings [1]. Leisure ocean cruises provide an ideal setting for efficient transmission of SARS-CoV-2, given the socially dense exposure environment. While outbreaks have also occurred on military and other vessels [2], leisure cruise ships have been the most conducive for outbreaks of a variety of infectious respiratory and diarrheal diseases such as influenza and norovirus [3-5]. Because they represent a EUR 125 billion industry and provide many jobs globally [6], cruise ships are clearly vulnerable to pandemics.

The most publicised outbreak of coronavirus disease (COVID-19) on a cruise ship as at the time of writing (May 2021) was the Diamond Princess outbreak in Yokohama, Japan in early February 2020. This case garnered great attention early on in the pandemic. Of the 3,711 passengers on board, 712 tested positive for SARS-CoV-2; 311 of those who tested positive were asymptomatic for COVID-19 at the time of testing and nine died [7]. After multiple outbreaks were identified on other cruise ships, a No Sail Order was implemented by the United States (US) Centers for Disease Control and Prevention (CDC) on 14 March 2020 [8]. Even after the No Sail Order took effect, intermittent cases were reported among crew who remained on the ships. Cruises restarted in force over the summer of 2021 with different infection prevention strategies, frequent testing strategies and vaccination requirements [9,10].

Previous systematic reviews of the transmission of SARS-CoV-2 and response to COVID-19 outbreaks on cruise ships have been limited to searches of the scholarly literature and, therefore, reported only on a limited number of outbreaks covered in the scientific literature [11]. Understanding the dynamics of outbreaks on cruise ships and evaluating the non-pharmaceutical interventions that have been successfully or unsuccessfully employed on these ships can inform future prevention research and policies, as well as benefit planning for future outbreaks with similar transmission patterns.

In this review, we evaluated PubMed and news sources to collate information on COVID-19 outbreaks on cruise ships before 1 October 2020, along with measures taken and clinical outcomes. The objectives of the study were to (i) determine the number of commercial ocean line cruise ships associated with COVID-19 cases before 1 October 2020 and (ii) characterise these outbreaks based on number of cases, number of passengers (both crew members and guests), number of people at risk, timing of the outbreak, number of COVID-19 diagnostic tests performed and clinical outcomes.

## Methods

### Information sources

We searched for data on COVID-19 outbreaks associated with ocean cruise ships via PubMed for case reports, brief reports and reviews [12]. To include cruise ship outbreaks not described in the biomedical literature, news sources were searched as well. The database compiled by the Miami Herald [13] of news reports and the list of cruise ships reporting COVID-19 outbreaks provided by the US CDC [14] were used as foundational material. All sources were checked and expanded upon via Google News searches of the cruise ship name and keywords (see below). The articles were then read and assessed for information about number of cases, outcomes of cases, number of crew members and guests at risk, as well as location of the outbreak, and these data were stored in an Excel spreadsheet. A review of articles retrieved from PubMed and newspaper articles was performed by two authors (KW and LP). We contacted the articles’ authors and the cruise lines for clarification, as needed [15].

### Search strategy

Search terms PubMed were (‘COVID’ OR ‘COVID-19’ OR ‘coronavirus’ OR ‘SARS-CoV-2’ OR ‘SARS’) AND (‘ship’ OR ‘ships’ OR ‘cruise’ OR ‘cruises’ OR ‘liner’) from 1 November 2019 to 1 October 2020. The Miami Herald database [13] of news reports and the US CDC list of cruise ships reporting COVID-19 outbreaks were searched, and checked against Google News searches of the cruise ship name and keywords. The search was completed on 5 October 2020. Irrelevant articles that did not address cruise ships and COVID-19 were excluded by title and abstract review. Articles were included if they were published between 1 November 2019 and 1 October 2020 and described cases or clusters of COVID-19 on ocean line cruise ships among either guests or crew, or COVID-19 cases that were subsequently linked to these ships. The status of cases – both those who met the clinical criteria for COVID-19 but did not receive a test and those who had a positive SARS-CoV-2 test (test type was not often specified) were recorded as probable or confirmed COVID-19 cases as stated by authors of each respective article. Articles were excluded if they did not provide any specific information on the timing of the outbreak, number of passengers on board, number of cases or number of tests. River cruises, cargo ships and freight ships were excluded. Articles were excluded if they were commentaries without primary data. If non-English sources were referenced in other included sources, these were translated and included in the review, though non-English literature was not specifically searched.

### Definitions

A ‘cruise ship’ was defined as a given vessel or boat, while a ‘voyage’ was defined as a trip that the ship could take i.e. one ship could have multiple voyages. ‘Passenger’ was used to refer to both guests and crew members; when source information was not clear, individuals were counted as passengers. A COVID-19 outbreak was defined as at least one case of COVID-19 associated with a cruise ship voyage within 14 days of case disembarkment or at least one case of COVID-19 associated with guests or crew still on board, according to the definition of the US CDC at the start of the pandemic [16]. The resulting list of cruise ships was cross-referenced with the US CDC’s list of international cruise ships with COVID-19 outbreaks [14] and cases were verified through CruiseMapper, (https://www.cruisemapper.com/accidents ) and WikiWand (https://www.wikiwand.com/en/COVID-19_pandemic_on_cruise_ships ). Itineraries and locations were verified on several common, publicly available cruise websites, including CruiseMapper (https://www.cruisemapper.com ), Crew Center (https://crew-center.com ) and Iglu Cruise (https://www.iglucruise.com ). The number of guests and crew members were also verified on Crew Center or alternative sources if the cruise was not on this website. Location of the outbreak – when unclear – was noted as the next port of call. Because international ships report their occupancy to the US Securities and Exchange Commission (SEC; https://www.sec.gov), this information was used to estimate the number of passengers for the first two quarters of 2020. If 2020 reports were not available, the 2019 reports were used.

### Analysis

A descriptive analysis was performed characterising the COVID-19 cases. Cruise ships were stratified into cruise voyages with less than 20 cases, 20 to 100 cases and greater than 100 cases. A sensitivity analysis was performed including voyages with two or more cases identified. A narrative review was also performed for the cruise ships with more than 100 cases. Data analysis was done in RStudio (version 1.3.1056; R version 4.0.2, Vienna, Austria). When the number of guests or crew on board was not explicitly stated, the number of passengers was estimated from SEC reports.

## Results

### Literature review

**Figure 1:**
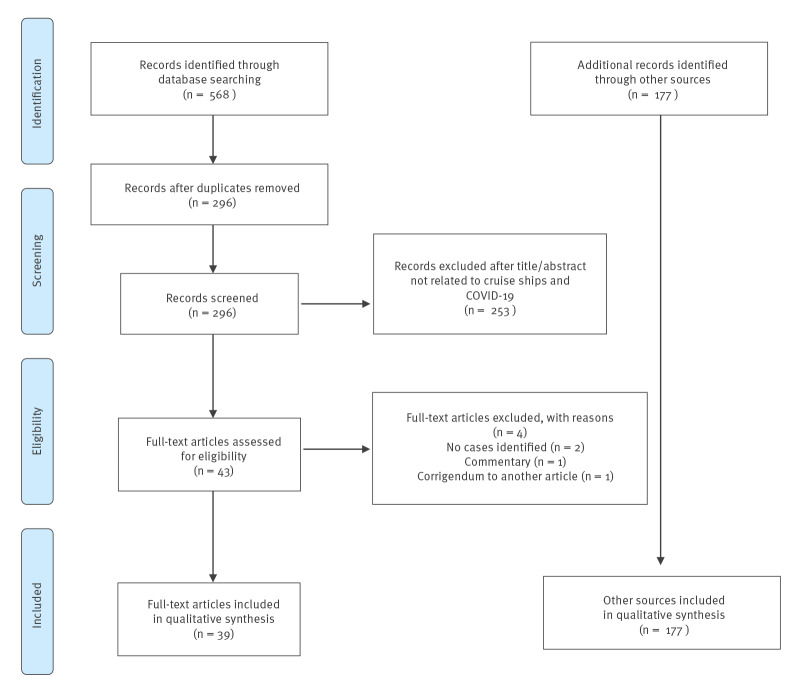
PRISMA flowchart for search strategy and literature review of COVID-19 cases on cruise ships worldwide, November 2019–October 2020

A total of 79 ships and 104 unique voyages were associated with COVID-19 cases before 1 October 2020. These ships were cross-referenced against the US CDC’s list of international cruise ships associated with COVID-19 outbreaks; no additional ships were found. From the PubMed search, 568 papers were identified. After removal of duplicates, as some papers had different identification numbers on preprint servers and journal websites, 296 papers were identified; however, only 43 were directly related to cruise ships based on their titles and abstracts, and were included for further analysis (Figure 1). After a review of each full paper from PubMed, 39 papers were included. Of those, 30 papers involved the Diamond Princess and six papers addressed cruise ships docked in Australian waters (though the number, names and voyages were not provided in the reports). One article about the public health response to the Diamond Princess also reported on the Grand Princess. The Ruby Princess, the Greg Mortimer and the Costa Atlantica each had one article. For the remainder of the 74 ships, all information came from a total of 177 news articles). The occupancy data from the SEC can be found in the supplement (Table S1).

COVID-19: coronavirus disease; US CDC: United States Centers for Disease Control and Prevention.

Records of biomedical literature, including case reports, brief reports and reviews, were identified through database searches in PubMed. Additional sources used included the database compiled by the Miami Herald [13] of news reports and the list of cruise ships reporting COVID-19 outbreaks provided by the US CDC were used as foundational material. All sources were checked and expanded upon via Google News searches of the cruise ship name and keywords.

### Descriptive analysis of cruise ships

The number of ships associated with COVID-19 cases was distributed across a variety of cruise lines. Twenty-four of 51 different cruise line companies had at least one case of COVID-19. Overall, 22.3% (79/354) of ocean line cruise ships had at least one case of COVID-19 from January to October 2020 [14].

Nineteen ships had more than one voyage with a case of COVID-19. Of these, 14 had an increase in the number of cases in later voyages. Four of the 19 cruise ships had one case reported for each voyage. One ship of the 19 had a decrease in COVID-19 cases over time (the Costa Favolosa had a decrease in cases from 58 of 4,789 passengers to 2 of 1,009, the later outbreak was when only crew was on board and with one fifth the number of passengers; Supplementary Table S2).

There was sparsity of data for many of the 104 voyages; 21 voyages only reported one case and no associated date, of these, 11 voyages from 10 ships only reported one case and no other information was available about these cruise ships, such as if guests or crew were infected. These voyages were on the list of cruise ships from the US CDC.

Only 18 of the 104 voyages provided information about the number of COVID-19 tests that were performed and 10 of these voyages had only crew on board. These voyages that provided SARS-CoV-2 PCR testing were later in the year than those that did not provide testing, with a mean date of outbreak identification on 2 April 2020 (standard deviation (SD): 27.8 days) compared with 9 April March 2020 (SD: 42.9 days) for those that did not provide testing. Of these 18 voyages, a median of 234 tests were performed (IQR: 30–939), which covered 45.5% of all cruise ship passengers (11,141/24,492). For six cruise voyages, 100% of the passengers were tested, with a median number of 127 COVID-19 cases (IQR: 67–144) identified and a median attack rate of 37.0% (IQR: 21.1–56.8).

The median total number of passengers at the time of outbreak was 2,040 passengers (IQR: 969–4,269). However, this is a rough estimate, as a confirmed value was only available for 44 of the 104 voyages and the remainder were estimated based on cruise ship capacity. The median number of days from when passengers disembarked to identification of the outbreak was 7 days (IQR: 2–12). However, there was a large proportion of missing data for this calculation, with only 56 of 104 voyages included.

Examining all voyages, the number of cases and deaths were low. The median number of cases per ship was three (IQR: 1–18), with two notable outliers: the Diamond Princess and the Ruby Princess, which had 712 and 907 cases, respectively. The median overall attack rate was 0.2% (IQR: 0.0–1.5), though this distribution was skewed to the right with a mean attack rate of 3.8%; 25.9% of voyages had at least one COVID-19-associated death. The median number of COVID-19-associated deaths across those ships with cases was zero (IQR: 0–1), though again there was a right-skew of the data. Three voyages had a 100% CFR; however, each of these voyages only reported one case (Supplementary Table S3).

The sensitivity analysis showed 65 voyages were associated with two or more COVID-19 cases; of these, the median number of cases per ship was higher compared with all voyages (n = 12; IQR: 4–48), with a median attack rate of 0.9% (IQR: 0.39–3.5). The median number of deaths in this sensitivity analysis was still zero (IQR: 0–1).

### Analysis by outbreak size

To gain a greater understanding of outbreak characteristics based on outbreak size, the cruise ship voyages were divided into three groups based on number of COVID-19 cases: less than 20 cases (n = 78), 20 to 100 cases (n = 17) and greater than 100 cases (n = 9). All voyages associated with a particular ship are presented (Table 1 and Supplementary Table S2 and S3); each voyage was analysed with its respective number of cases, e.g. Voyage A of Ship X, with 120 cases, is included as ‘greater than 100 cases’ and Voyage B of Ship X, with 50 cases, is included as ‘20 to 100 cases’. Nine voyages had greater than 100 cases; these outbreaks occurred earlier in the pandemic, with the median outbreak identified on 24 March 2020 (SD: 27.7 days) (Table 1). Five of these large outbreaks happened with passengers on board, i.e. Ruby Princess, Diamond Princess, Ovation of the Seas, Grand Princess, Greg Mortimer. The remaining four had only crew on board, i.e. Disney Wonder, Costa Atlantica, Celebrity Apex, Horizon, and occurred several weeks later (median date: 7 April 2020; IQR: 23 March–26 April 2020). The median attack rate for these voyages was 21% (IQR: 19.2 –23.9) and CFR was 0.8% (IQR: 0–2.0). Two ships had an attack rate of over 50%: the Greg Mortimer, which had an attack rate of 59% with both guests and crew on board, and the Horizon, with an attack rate of 50% and only crew on board. The Grand Princess had the highest CFR of 4.1% while the Ruby Princess had the highest absolute number of deaths of passengers (n = 29), of which 21 were guests. Even in these outbreaks with a higher number of COVID-19 cases, there was often still uncertainty about data quality and completeness; for example, the Disney Wonder reported ca 200 crew cases, though no further specific details about this outbreak could be obtained.

**Table 1:** Cruise ships with any voyage over 100 cases of COVID-19, 3 February 2020–12 May 2020 (n = 9)

Cruise ship	Travel event date (2020)			Location of ship when outbreak identified	Number of passengers at time of outbreak			Number of SARS-CoV-2 PCR tests performed	Known cases			Known deaths			Attack rate	Case Fatality rate
	Embarkment	Passenger disembarkment	Outbreak identified		Total	Guests	Crew		Total^a^	Guests	Crew	Total	Guests	Crew		
Ruby Princess	8 Mar	19 Mar	20 Mar	Sydney, Australia	3,795	2,647	1,151	NA	907	605	202	29	21	0	23.9	3.2
Diamond Princess	20 Jan	Variable	3 Feb	Yokohama Port, Japan	3,711	2,666	1,045	3,618	712	554	152	14	8	0	19.2	2.0
Celebrity Apex	> 14 days without passengers	NA	25 Mar	Santi-Nazaire, France	1,440	0	1,440	1,440	284	0	284	0	0	0	15.4	0.0
Disney Wonder (Voyage A)	28 Feb	2 Mar^b^	NA	NA	4,260^b^	3,325^c^	935^b^	NA	1	0	0	0	0	0	0.0	0.0
Disney Wonder (Voyage B)	6 Mar	19 Mar	29 Mar	San Diego, CA, US	1,980	1,045	935	NA	36	3	33	1	1	0	1.8	2.8
Disney Wonder (Voyage C)	> 14 days without passengers	19 Mar	12 May^b^	NA	935	0	935	NA	200^b^	0	200	0	0	0	21.4	0.0
Costa Atlantica	> 14 days without passengers	NA	21 Apr	Nagasaki, Japan	623	0	623	623	149	0	149	0	0	0	23.9	0.0
Greg Mortimer	15 Mar	28 Mar	24 Mar	Montevideo, Uruguay	217	132	85	217	128	0	37	1	0	1	59.0	0.8
Horizon	> 14 days without passengers	15 Mar	26 Mar	Dubai, UAE	250^b^	0	250^b^	250^b^	125^b^	0	125^b^	0	0	0	50.0	0.0
Grand Princess (Voyage A)	11 Feb	21 Feb	4 Mar	Oakland, CA, US	4,200^b^	3,100^b^	1,100^b^	NA	21	21	0	2	2	0	0.5	9.5
Grand Princess (Voyage B)	21 Feb	Variable	4 Mar	Oakland, CA, US	3,533	2,422	1,111	1,103	123	104	19	5	4	1	3.5	4.1
Ovation of the Seas	12 Mar	18 Mar	20 Mar	Sydney, Australia	5,300^b^	3,800	1,500^b^	NA	109	107	0	1	1	0	2.1	0.9

CA: California; COVID-19: coronavirus disease; NA: not available; SARS-CoV-2: severe acute respiratory syndrome coronavirus 2; SEC: Securities and Exchange Commission; UAE: United Arab Emirates; US: United States.

^a^ Total COVID-19 cases may not equal the sum of crew and guest cases because some reports did not identify cases as crew members or guests.

^b^ Numbers are approximate; when number of guests was not known, the maximum occupancy of guests was used.

^c^ Numbers were taken from SEC reports.

Of the 17 ships that had one voyage with 20 to 99 cases of COVID-19 (Supplementary Table S2), six only had crew on board and the median number of cases was 44 (IQR: 27–58), the median attack rate was 2.9 (IQR: 1.2–6.4) and the CFR was 2.6% (IQR: 0–5.0). Mean date outbreak identified 13 March 2020 (SD: 35.8).

The remaining 61 ships and 78 voyages had less than 20 cases (Supplementary Table S3). The mean date of outbreak identification was 2 March 2020 (SD: 32.9 days). Twenty-seven of the 78 voyages had only crew on board. These 61 ships had a median of two COVID-19 cases (IQR: 1–4). The median attack rate was the lowest of the three groups at 0.1 (IQR: 0.0–0.3). However, for three ships that had only crew on board, the only case reported was a fatality.

**Figure 2:**
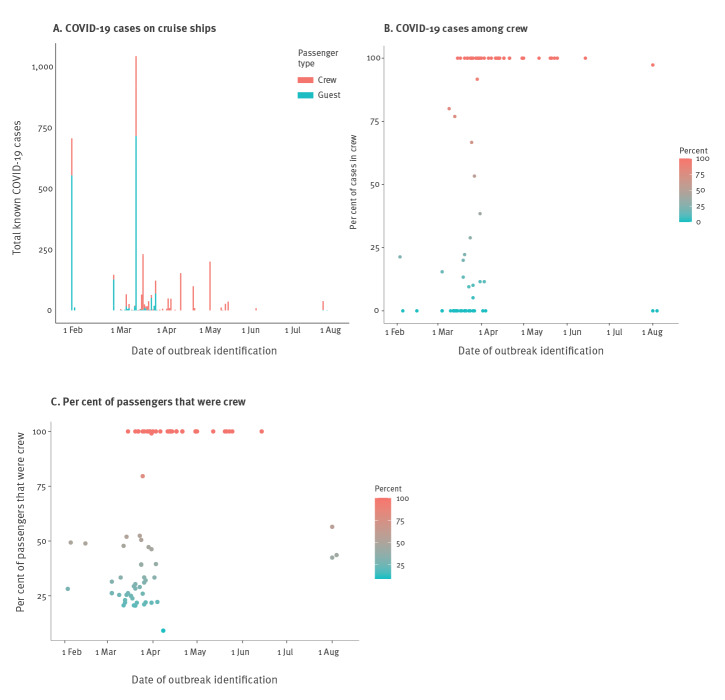
Number of COVID-19 cases in guests (n = 1,658) and crew members (n  = 1,792) on cruise ships over time, worldwide, 1 February–1 August 2020

Thirty-seven of the 104 total voyages had only crew on board during the COVID-19 outbreak, with a median of seven cases (IQR: 2–27). These voyages occurred later than those with guests and crew on board (Figure 2). Of these voyages, eight (21.6%) reported deaths. The resulting median attack rate was 0.5 (IQR: 0.2–5.6) although, as mentioned previously, for three ships the only case reported was a fatality. The Celebrity Flora had the highest attack rate of any of the ships, at 69.6%. All 69 crew members were tested for SARS-CoV-2 and 48 tested positive, although no fatalities were reported. Similarly, on the Horizon, all 250 crew members were tested with a resulting attack rate of 50%. The Celebrity Apex, which was in France preparing for its maiden voyage and only had crew on board, had an attack rate of 19.7% and 100% of the crew members tested; no deaths were reported [17].

COVID-19: coronavirus disease.

### Narrative review

To offer a clearer picture of different cruise ship scenarios, we have produced a chronological, narrative review of cruise ship voyages with over 100 COVID-19 cases. The first four voyages are described in the main text, and descriptions about the other ships (n = 6 of 9 voyages) can be found in the Supplement.

#### Diamond Princess – departed from Yokohama Port, Japan, 20 January 2020

The Diamond Princess cruise ship has 1,337 passenger cabins and can hold a maximum of 2,670 guests and 1,100 crew members [7,18-20]. The outbreak on the Diamond Princess was the first COVID-19 outbreak identified on a cruise ship. At the time of the outbreak, there were 2,666 guests and 1,045 crew members on board [7,18-20]. On 25 January 2020, 5 days after the ship’s departure from Yokohama Port, Japan, a guest with upper respiratory symptoms disembarked in Hong Kong. On 1 February, the guest tested positive for SARS-CoV-2. Ten additional cases were identified on 4 February after the Japanese Ministry of Health conducted initial screening tests to identify further cases on the ship. All guests on board were placed into quarantine on the ship in Yokohama Port for 14 days. Guests at highest risk for severe disease were tested and repatriated before the end of the 14-day quarantine. Guests and crew who tested positive were removed from the ship and taken to a hospital on land [7,18-20]. Guests who remained on board were required to isolate in their rooms, but were allowed into common areas during specified time windows if they wore masks and gloves. Crew members continued to live in their quarters, ate in common areas and were required to wear personal protective equipment (PPE), i.e. face masks [21] and gloves [22], outside of their cabins [7,23-25]. Crew members also delivered food to guests in pairs of two, wearing full PPE. Following the repatriation of all guests, the crew completed an additional 14-day quarantine in the guest cabins. In total, there were 712 cases, 554 among the guests and 152 among the crew [7,18-20].

As this outbreak occurred early on in the COVID-19 pandemic, studies on the Diamond Princess outbreak provided foundational knowledge about the clinical course, testing characteristics, epidemiology and utility of infection control practices when there was little other biomedical literature available.

#### Grand Princess - Voyage A departed from San Francisco, CA, 11 February 2020; Voyage B departed from San Francisco, 21 February 2020

The Grand Princess cruise ship can hold around 3,100 guests and 1,100 crew members. COVID-19 cases were identified on two Grand Princess voyages, which have been widely referred to as ‘Voyage A’ and ‘Voyage B’. Voyage A departed San Francisco, California (CA), US on 11 February 2020, sailed to Mexico and then returned to San Francisco on 21 February [7,26]. Some guests and crew members remained on the ship for Voyage B, which left San Francisco for Hawaii on 21 February, carrying 2,422 guests and 1,111 crew members. On 4 March 2020, COVID-19 cases were identified in guests and crew from Voyage A, prompting testing on Voyage B [7]. The ship was quarantined in Oakland, CA. However, during the quarantine, only 1,103 tests were performed (type of test not specified), because of concern among the guests that testing positive would require them to quarantine for longer on land [27,28]. Ultimately, 123 cases were identified and five people died – four guests and 1 crew member [7]. Because only around 30% of guests and crew were tested, the true number of cases may have been higher than reported.

#### Ruby Princess – departed from Sydney, Australia, 8 March 2020

The Ruby Princess cruise ship departed Sydney, Australia on 8 March 2020, with ca 2,647 guests and 1,151 crew members. Throughout the course of the 11-day voyage, over 100 guests presented to the ship’s medical centre for upper respiratory symptoms; 36 guests presented to the medical centre with influenza-like symptoms, accounting for 0.94% of the ship’s total passengers. As the prevalence of influenza-like-illness did not reach the 1% threshold for an outbreak set by the New South Wales Ministry of Health, the ship was classified as ‘low-risk’ as opposed to ‘high-risk’ [29]. The situation was further complicated, as some rapid influenza tests run aboard the Ruby Princess came back positive for influenza A. However, many patients presenting with influenza-like symptoms tested negative. Upper respiratory samples were sent to a testing facility in Sydney, as the ship did not have the capacity to test for SARS-CoV-2 (test type unclear). Later reporting revealed that 120 people on board the Ruby Princess met the case definition for COVID-19 at the time of disembarkment [30]. Passengers were allowed to disembark in Sydney on 19 March. On the same day, the first passenger cases were identified [29]. In total, 907 primary cases were identified (605 guests and 202 crew members) and 29 people died. In late April, the Ruby Princess outbreak was linked to 13% of all COVID-19 cases in Australia [31].

## Discussion

SARS-CoV-2 falls into a class of pathogens that spreads readily on cruise ships, such as influenza and norovirus [32,33]. These pathogens can spread quickly when many people are in close proximity for prolonged periods of time, and rapid action is required to prevent larger outbreaks. As opposed to the spread of COVID-19 in indoor settings, where cluster size is usually around five people per household [34,35], cruise ship environments have the potential for case numbers to rise to several hundreds. A review of multiple superspreading events of COVID-19 indicates that 40% of published COVID-19 superspreading events are associated with travel [36]. Closed environments are associated with 18.7 times greater odds of transmitting SARS-CoV-2 from a primary to a secondary case compared with open-air environments [37] and cruise ships – by design – have extensive, heavily trafficked, closed environments.

COVID-19 outbreaks on cruise ships have the potential to accelerate international spread of the disease. Several of the larger outbreaks (greater than 100 cases per voyage) were associated with subsequent superspreader events once passengers returned to their countries of origin, which was particularly notable early in the COVID-19 pandemic. For example, over 10% of COVID-19 cases in Australia were linked to the Ruby Princess and two of its passengers were the likely source of a major outbreak in Tasmania [38], while 16% of the COVID-19 cases in the state of Iowa, US were linked back to the MS Asara (a river cruise) in Egypt [39]; 17% of all US cases early in the pandemic were linked to cruise ships [7]. Cruise ships also brought the first cases of COVID-19 to islands such as St. Lucia, Cuba, the Cayman Islands and Puerto Rico [17]. Of the 79 cruise ships and 104 voyages identified in this study, the median attack rate for COVID-19 was 0.2% (IQR: 0.03–1.5), but there was significant heterogeneity in the attack rate, which ranged from 0.1% to 69.6%. This estimation is limited by the large amount of missing and estimated data throughout the dataset. However, understanding how COVID-19 can potentially spread on cruise ships will be important to prevent future outbreaks.

The number of COVID-19 cases associated with cruise ship voyages during the early COVID-19 pandemic spanned a large range, from one to 907 cases. Cruise ship-associated outbreaks could largely be divided into four notable groups: (i) large outbreaks (greater than 100 cases) that were heavily reported in news media and for which more complete though not authoritative information was available (Table 1); (ii) outbreaks with less than 100 cases; (iii) outbreaks for which there was limited information other than that a case was associated with a cruise ship voyage within 14 days of disembarkation; and (iv) outbreaks among crew members after guests disembarked.

It was often not known how many COVID-19 tests were obtained on board the cruise ships; only 19 of the 79 ships reported this number. SARS-CoV-2 PCR testing capacity was limited early on in the pandemic, which could account for the low testing rates and the low reporting of test numbers at that time. Of the 19 ships that reported the number of tests performed, six (five of which had only crew on board) tested 100% of people on board; as these ships had a high median attack rate of 40%, this suggests that if further testing had been performed on other voyages, more cases may have been found.

Another important theme that emerged in this review was the vulnerability of the crew. After guests disembarked following the US CDC’s No Sail Order passed on 14 March 2020, a considerable number of crew members remained on board the cruise ships for prolonged and undetermined amounts of time and under difficult conditions [40,41]. Three ships with only crew on board each reported one case of COVID-19 that was a death. As the CFR is estimated to be between 1.2 and 4.7% in the general population [42], these ships with only one fatal case suggest under-reporting of the total number of cases. Finally, COVID-19 was not the only illness that had a profound impact on the health of the crew, as the psychological toll of the pandemic and working conditions during this period should not be underestimated.

Europeans are the second largest group of cruise ship passengers globally: in 2019, over 7.7 million Europeans travelled on cruises and 7.6 passengers embarked from European ports [43]. In addition, Europe is the second most common region of cruise ship deployment after the Caribbean, with 28% of the global industry deployed from Europe, 17% of which is based in the Mediterranean. Several cruise companies are based in Europe, including MSC Cruises, which carried 2.7 million passengers in 2019, and TUI Cruises, which carried 1 million passengers in 2018–19. Carnival Cruises, which has the capacity to carry 3.2 million passengers from the European market, is British- and American-owned. The number of passengers on cruise ships has begun to increase again in 2021, after a nadir in 2020 [14]. Over the summer of 2021, cruises have restarted in Europe with strict non-pharmaceutical measures including distancing and testing, as well as vaccination requirements in place.

### Limitations

The search for epidemiological information on SARS-CoV-2 infections was limited by several factors. For 67 of the ships included in this study, all the epidemiological information came from news reports rather than the scientific literature. News and media sources are infrequently the primary source for scientific publications, and not all cases of COVID-19 on cruise ships might have generated news articles. For example, earlier in the pandemic, cases remotely associated with a cruise ship might have been reported, but later in the pandemic—as the virus was more widespread throughout multiple communities—such pieces might not have been reported. Crew members or guests with easier to access to reporters, or cruise ships closer to large cities might be more likely to be featured in a news report. It would be beyond our ability to fully classify this publication bias. Furthermore, news articles often do not completely report all the epidemiological information typical for a scientific publication of an outbreak. News sources might have been published in the middle of an outbreak, as opposed to at the end of an outbreak, so final numbers may not be accurate. As data were scarce, any epidemiological information contained in the articles was included, which may contribute to some lower quality of evidence in the source material. Considering all data obtained in this study, limitations also include a large amount of missing data on multiple variables. For example, only 19 voyages reported on the number of SARS-CoV-2 tests performed on board. Eleven cruise ships were only identified via the US CDC list of cruise ships, but no further information was found, resulting in substantial epidemiological gaps. These voyages were counted as having only one case. When not explicitly reported, the number of guests was estimated using the maximum number of guests, which may also have underestimated them attack rate. At times, we identified conflicting reports, such as a crew member reporting that tens to hundreds of crew members had viral upper respiratory tract infections, but no cases were confirmed by the cruise line [25,44]. On the Norwegian Gem, three deaths were reported, including the cruise doctor; however, no COVID-19 cases were reported, although tests were available [45].

Very few ships, primarily those with over 100 cases, had extensive epidemiological characterisation of their outbreaks. In ships with lower case numbers, vital epidemiological information was often difficult to determine, such as when the outbreak was identified, how many people were at risk and how many tests were performed. Only the Diamond Princess, the Grand Princess and the Greg Mortimer had thorough epidemiological descriptions published in the scholarly literature. The remainder of the outbreaks were defined primarily on information from news sources. We performed a parallel search for ‘COVID-19’ or ‘SARS-CoV-2’ and ‘cruise’ or ‘cruise ship’ using Nexis Uni (https://www.lexisnexis.com/en-us/professional/academic/nexis-uni.page) yielded over 10,000 results. This was beyond our capacity to review in full, but a randomly selected subset of results did not suggest any relevant information. Therefore, this database was not included in this study as a source of information. However, the news citations included in our search were the top results from the Google News search engine, and therefore some cases or reports could have been missed. Reassuringly, a meta-analysis of the scholarly literature by Kordsmeyer et al., which examined four databases over a shorter duration (January–July 2020) was roughly in line with the observations of our search [11]. In this study, a total of 37 studies were included, of which 33 reported outbreaks of SARS-CoV-2 on cruise ships (27 of these studies referred to the Diamond Princess). Two studies considered outbreaks on the Grand Princess, three studies informed about Nile River cruises (which was excluded from our analysis) and one study about the MS Westerdam (which may have had multiple outbreaks).

With the re-launching of cruises in 2021, the European Centre for Disease Prevention and Control and the US CDC proposed updated guidance for risk mitigation [8,46]. Strategies to mitigate risk at the individual level may include mandatory COVID-19 testing before embarkation, temperature checks and health questionnaires before disembarkation and intermittently throughout the cruise, as well as recommendation/verification of vaccination of guests and crew, a pre-travel negative test, or proof of recovery from COVID-19 before travel, although protocols may vary by cruise company and port of departure [47-49]. Other proposed control measures include managing the number and flow of passengers to allow for physical distancing, mandatory mask wearing while on board and contact tracing for positive cases. Environmental upgrades include updating heating, ventilation and air conditioning (HVAC) systems to include high efficiency particulate air (HEPA) filters in high-risk locations; increased frequency of environmental cleaning; and greater access to hand sanitizer to decrease potential transmission [50,51]. A registry of guests and crew with comorbidities and medications could also be helpful for triage and resource allocation if passengers were required to stay on board during an outbreak. Modelling of passenger movement could also be used to help model maximum occupancy, as has been done in other settings [52,53]. Each cruise ship should have proposed policies or plans in place for assessing the COVID-19 epidemiology of destinations, plans for medical evacuation and repatriation of guests and outbreak contingency plans [46]. With the rise of the omicron variant, as of December 30, 2021 the US CDC considers cruise ship travel Level 4 risk and recommends avoidance of all cruise ship travel regardless of a person’s vaccination status [54].

Because of the limitations of the data presented, a centralised global registry similar to the US CDC norovirus registry would be useful [55]. Several countries, such as Jamaica [56], New Zealand [57] and Australia, have produced some reports for cruise ships in their waters, but there is no international registry of cruises. A consolidated registry would allow for all information to be present in a single location with minimum quality and standard data that would allow for future meaningful epidemiological studies of COVID-19 on cruise ships and a greater understanding of the disease overall [55]. In the interim, we have collated the available data so that they can be used for analysis and to improve understandings of COVID-19 outbreaks on ships.

### Conclusions

COVID-19 can spread easily on cruise ships because of the confined space and high density contact networks, which can create superspreader events. As has been shown by previous analysis, considerable measures need to be taken when cases are identified on a cruise ship, such as immediate isolation of cases upon diagnosis, restriction of guests to cabins, limiting guest and crew contacts and performing early mass screening with a high sensitivity rapid tests. It is important to have accurate record keeping of the number of cases and the interventions implemented so that it is possible to properly track the situations that are most prone to COVID-19 outbreaks and to understand which interventions provide successful mitigation. As cruise ships continue to resume their operations, these steps remain important to prevent outbreaks.

## Statements

**Ethical statement:** Institutional Review Board approval was not sought as all data were gleaned from public sources such as scientific or news articles.

**Funding statement:** This study was supported with funding from the Yale Institutes for Global Health. This work was funded in part by National Institutes of Health Grant 5T32AI007517–20 (to L.P.)

## Supplementary Data

1176000 Supplement
