# Supplementary material for: A review of COVID-19 transmission dynamics and clinical outcomes on cruise ships worldwide, January to October 2020
Source: Euro Surveill. 2022 Jan 6;27(1):2002113. doi: 10.2807/1560-7917.ES.2022.27.1.2002113 (PMC8739343; doi:10.2807/1560-7917.ES.2022.27.1.2002113)
Supplement: Supplement [file 20-02113_WILLEBRAND_supplement.pdf]

SUPPLEMENT

Disclaimer: This supplementary material is hosted by Eurosurveillance as supporting information alongside the article " on behalf of the authors who remain responsible for the accuracy and appropriateness of the content. The same standards for ethics, copyright, attributions and permissions as for the article apply. Eurosurveillance is not responsible for the maintenance of any links or email addresses provided therein

Supplement 1: Narrative review of the remaining 9 ships.

Ovation of the Seas (Departed from Sydney, Australia on 12 March 2020)

On March 12, 2020, the Ovation of the Seas left Sydney, Australia for a round-trip voyage to New Zealand carrying roughly 3,800 people. However, due to the COVID-19 pandemic, the ship was refused entry to port in New Zealand. The ship returned to Sydney on March 18th without docking anywhere else and all passengers were allowed to disembark. The first positive case linked to the Ovation of the Seas was identified on March 20[1]. Guests were notified of their potential exposure via an email sent on March 22. 109 people, including 107 guests and 2 crew members, became sick and 1 guest died.

Greg Mortimer (Departed from Argentina on 15 March 2020)

The Greg Mortimer departed from Argentina on 15 March 15 2020, for a planned 16-day trip to Antarctica and South Georgia with 132 guests and 85 crew on board[2]. However, the ship was forced to divert course for Uruguay after several passengers, including the ship doctor, presented with symptoms of COVID-19. The ship was quarantined in Uruguay. 128 of the 217 passengers tested positive. Eight passengers required hospitalization.

The outbreak on the Greg Mortimer cruise ship offers a unique opportunity for modeling, as all

guests and crew members were tested. A detailed report of the incident was published in BMJ by Ing et. al[3].

Celebrity Apex (Docked in Saint-Nazaire, France)

The Celebrity Apex was docked in Saint-Nazaire, France, with 1407 crew on board. Starting 7 March 2020, the crew were required to remain on the ship. However, multiple statements from crew members report that contractors working on the ship were not restricted from leaving and re-entering the ship[4].

On March 21st, Celebrity Cruises hosted a large in-person training for crew members, followed by a company-hosted party. Crew members report being encouraged to attend by Celebrity executives[4]. On March 23rd, the first case of COVID-19 was identified on board. On March 25th, crew members were given PPE to be worn outside their cabins. Ultimately, out of the 1,407 crew members onboard, 217 people tested positive. There were no deaths reported from this outbreak.

Pullmantur Horizon (Docked in Dubai, UAE)

Passengers disembarked the Pullmantur Horizon on 15 March 2020 and the ship was docked in Dubai, UAE. Crew reported that they were notified on 28 March 2020 that crew members had tested positive on March 26th after being taken off the ship to a local hospital. Crew was separated into two areas on the ship. On April 4th, crew reported 150 cases out of 250 people

on board the ship. Information regarding this outbreak is limited, as all information was provided by anonymous crew members to Crew Center[5].

Costa Atlantica (Docked in Nagasaki, Japan)

Like the Celebrity Apex, the Costa Atlantica was not hosting guests at the time the COVID-19 outbreak was identified. In January, the ship docked in Nagasaki, Japan for repairs. 623 crew remained onboard. Crew were not allowed to disembark the ship after the onset of the COVID-19 pandemic. On 21 April 21 2020, the outbreak was identified. It was later reported that crew members had left the ship to go into town, despite orders not to. Crew were locked down in the ship and widespread testing was rolled out. A total of 149 crew members tested positive for COVID-19 as a result of the outbreak. There were no deaths reported[6].

Disney Wonder, Carnival Valor, and Celebrity Eclipse: These three ships have been grouped as they are reported to have over 100 cases associated with them in total, though no single outbreak onboard was reported to be over 100 cases.

Disney Wonder: There were three voyages associated with COVID-19, embarking on the following dates: 28/2/2020, 6/3/2020, 19/3/20. On the March 6th voyage, concerns were raised about a crew member showing symptoms of COVID-19. However, the crew member tested positive for Influenza A and B, leading to a de-escalation in concern. According to the Miami Herald, 36 total people tested positive for COVID-19 after this voyage. However, reporting after the March 19th voyage brings the true number of cases into question. Anonymous crew members have reported that there were over 200 positive cases on board after this voyage. However, there are no public reports from

Disney Cruise Lines or public health agencies about the total number of cases on this ship. The Miami Herald reports a total of 260 COVID-19 cases - 229 among crew, 31 among guests - associated with the Disney Wonder.

Celebrity Eclipse: The Celebrity Eclipse disembarked from San Francisco, CA, USA on March 1st. Passengers report that the crew staff had concerns about passengers who were experiencing COVID-19-like symptoms at the beginning of the cruise. However, cruise activities proceeded normally until the CDC's No Sail order was passed on March 15th. After this date, the ship reversed course near Chile to return to port in the US. On March 21st, cruise management hosted an event to honor healthcare professionals working to combat the COVID-19 pandemic, crowding passengers and crew onto the ship deck. On March 30th, passengers disembarked in San Francisco[7]. It is unclear when the initial cases were identified, but one day after disembarking passengers were notified via email of potential exposures on the ship[8]. There were 78 cases reported to be associated with this outbreak, though the Miami Herald reports a total of 123 cases associated with this ship.

Carnival Valor: A single case was denoted by Miami Herald for each voyage, as the total number of cases per voyage could not be validated. They report 132 total cases associated with this cruise ship.

Supplemental Table S1: Occupancy of guests and crew of cruise ships

Ship	Double occupancy of guests	Maximum occupancy of guests	Total capacity of crew	Occupancy percentage by cruise company
Adventure of the Seas	3,114	3,807	1,185	103
Black Watch	807	868	330	*
Braemar	989	1,075	400	*
Carnival Freedom	2,974	3,710	1,150	103
Carnival Imagination	2,056	2,594	920	103
Carnival Valor	2,968	3,710	1,150	103
Carnival Vista	3,954	3,954	1,450	103
Celebrity Apex	2,918	3,373	1,320	103
Celebrity Eclipse	2,852	3,000	1,500	103
Celebrity Flora	120	120	50	103
Celebrity Infinity	1,950	2,449	1,000	103
Celebrity Reflection	2,852	3,000	1,500	103
Celebrity Silhouette	2,852	3,000	1,500	103
Celebrity Solstice	2,852	3,000	1,500	103
Celebrity Summit	1,950	2,449	1,000	103
Coral Princess	1,950	2,491	980	103
Costa Atlantica	2,114	2,680	920	103
Costa Deliziosa	2,260	2,712	1,050	*
Costa Fascinosa	3,012	3,780	1,000	103
Costa Favolosa	3,012	3,780	1,050	103
Costa Luminosa	2,838	3,570	1,000	103
Costa Magica	2,688	3,470	1,000	103
Costa Victoria	1,928	2,200	800	103
Crown Princess	3,088	3,800	1,200	103
Diamond Princess	2,646	3,100	1,100	103
Disney Wonder	1,942	3,325	945	*
Enchantment of the Seas	2,252	2,730	852	103
Explorer of the Seas	3,286	4,290	1,185	103
Fridtof Nansen	528	600	150	*
Grand Princess	2,600	3,100	1,100	103

**Greg Mortimer**	120	160	74	*
**Harmony of the Seas**	5,479	6,780	2,100	103
**Horizon**	1,506	1,828	620	*
**Liberty of the Seas**	3,798	4,960	1,360	103
**Majesty of the Seas**	2,350	2,767	912	103
**Marella Discovery 2**	1,830	2,196	735	*
**Marella Explorer 2**	1,912	2,669	780	*
**Mariner of the Seas**	3,344	4,000	1,200	103
**Mein Schiff 3**	2,506	2,700	1,000	*
**MS Marina**	1,258	1,447	800	*
**MS Paul Gaugin**	318	332	217	*
**MS Queen Victoria**	2,014	2,250	1,253	103
**MS Roald Amundsen**	530	530	151	*
**MSC Armonia**	1,566	2,065	760	112
**MSC Bellissima**	4,488	5,386	1,400	112
**MSC Fantasia**	3,274	3,959	1,325	112
**MSC Grandiosa**	4,888	6,334	1,700	112
**MSC Meraviglia**	4,488	5,386	1,400	112
**MSC Opera**	1,756	2,055	730	112
**MSC Orchestra**	2,550	3,013	987	112
**MSC Preziosa**	3,502	3,960	1,300	112
**MSC Sea View**	4,132	5,331	1,413	*
**MSC Splendida**	3,274	3,959	1,325	112
**MV Artania**	1,188	1,260	537	*
**Norwegian Bliss**	4,200	5,200	1,730	104
**Norwegian Breakaway**	4,028	5,088	1,590	104
**Norwegian Encore**	4,266	5,218	1,731	104
**Norwegian Gem**	2,394	*	1,070	104
**Norwegian Pride of America**	2,186	*	927	104
**Oasis of the Seas**	5,400	6,296	2,165	103
**Oceania Riviera**	1,258	1,300	800	*
**Ovation of the Seas**	4,180	4,905	1,500	103
**Pullmantur Monarch**	2,356	2,772	827	*
**Queen Mary 2**	2,695	2,695	1,253	*
**Rhapsody of the Seas**	1,998	2,416	765	103
**Ruby Princess**	3,080	3,782	1,200	103
**Sea Dream**	112	112	95	*
**Seven Seas Navigator**	490	490	345	(e

**Scarlet Lady**	2,770	2,860	1,160	*
**Silver Explorer**	144	173	111	*
**Silver Shadow**	388	466	302	*
**Sky Princess**	3,668	4,402	1,350	103
**Sun Princess**	2,022	2,272	900	103
**Symphony of the Seas**	5,518	6,370	2,390	103
**Vision of the Seas**	2,036	2,443	765	103
**Voyager of the Seas**	3,282	3,938	1,181	103
**Westerham**	1,964	2,362	820	*
**World Dream**	3,376	3,400	2,000	*
**Zaandam**	1,438	1,440	561	*

Supplemental Table S1: Occupancy by cruise ship; * unable to find [9-25]

Supplemental Table S2: Ships of 20 to 99 cases of COVID-19 from January 2020 to October 2020

Ship	Date Embarkation	Date Passenger Disembark-ment	Outbreak Identified	Location of Ship when Outbreak Identified	Number of Passengers at time of Outbreak	Number of Guests	Number of Crew	Number of COVID-19 PCR Tests Performed	Total Known Cases	Known Guest Cases	Known Crew Cases	Total Known Deaths	Known Guest Deaths	Known Crew Deaths	Attack rate	Mortality rate
Costa Luminosa	2/24/20	2/30/20	3/13/20	At Sea	4,570[†]	3,570[‡]	1,000[‡]	-	3	3	0	1	1	0	0.1	33.3
Costa Luminosa	3/5/20	3/10/20	3/13/20	Marseille, France	1,780	1,370	410	-	65	13	50	7	6	1	3.7	10.8
MV Artania	12/21/19	variable	3/26/20	Fremantle, Western Australia	1,609	1,072	537	-	89	6	9	4	3	1	5.5	4.5
MSC Seaview	>14 days no passengers	-	4/30/20	Santos, Brazil	615	0	615	-	86	0	86	0	0	0	14.0	0.0
Celebrity Eclipse	3/1/20	3/30/20[†]	4/3/20[†]	San Diego, CA, USA	3,800[†]	2,300[†]	1,500[‡]	-	78	69	9	2	2	0	2.1	2.6
Costa Favolosa *	3/9/20	3/19/20	3/26/20	Pointe-a-pitre, Guadalupe	4,789	3,780[‡]	1,009	-	58	4	3	4	0	0	1.2	6.9
Costa Favolosa *	>14 days no passengers	3/19/20	3/26/20	Miami, FL, USA	1,009	0	1,009	-	2	0	2	1	0	1	0.2	50.0
Voyager of the Seas	2/25/20	3/7/20	3/21/20	Sydney, Australia	5,038[†]	3,938[‡]	1,100[†]	-	1	1	0	0	0	0	0.0	0.0
Voyager of the Seas	3/7/20	3/18/20	3/31/20	Sydney, Australia	5,038[†]	3,938[‡]	1,100[†]	-	52	43	6	2	2	0	1.0	3.8
Costa Fascinosa **	>14 days no passengers	-	4/12/20	Port of Santos, Brazil	764	0	764	-	49	0	49	5	0	5	6.4	10.2
Celebrity Flora	>14 days no passengers	-	4/14/20	North Seymour Island, Galapagos Islands, Ecuador	69	0	69	69	48	0	48	0	0	0	69.6	0.0
Carnival Freedom	3/8/20	3/14/20	3/25/20	Galveston, TX, USA	4,430[†]	3,280	1,150[‡]	-	1	0	1	0	0	0	0.0	0.0
Carnival Freedom	>14 days no passengers	3/14/20	4/3/20	Galveston, TX, USA	790	0	790	400	44	0	44	0	0	0	5.6	0.0

**MS Roald Amundsen**	2/16/20	3/26/20	4/4/20	Chile	681†	530‡	151‡		2	2	0	0	0	0	0.29	0.00
**MS Roald Amundsen**	7/17/20	7/31/20	8/1/20	Norway	363	209	154	154	37	1	36	0	0	0	10.19	0.00
**Vision of the Seas *****	>14 days no passengers	-	5/25/20	St Vincent/ Grenada	356	0	356	356	36	0	36	0	0	0	10.1	0.0
**Adventure of the Seas**	>14 days no passengers	5/19/20	5/23/20	Trelawny, Jamaica	1,344	0	1,344	1,044	27	0	27	0	0	0	2.0	0.0
**MSC Splendida**	2/14/20	3/22/20	3/24/20	Marseilles, France	2,837	1,721	1,116	-	25	0	25	0	0	0	0.9	0.0
**Costa Victoria**	3/7/20	3/25/20	3/23/20	(At sea) returning to Italy from Mumbai, India	727	727	800‡	-	21	3	2	0	0	0	2.9	0.0
**Celebrity Solstice**	3/20/20	4/4/20	4/2/20	Sydney, Australia	4,500†	3,000‡	1,500‡	-	20	19	0	1	1	0	0.4	5.0

Supplementary Table S2: Cruises with between 20 and 99 cases of COVID-19; Red text = Approximate, when number of guests not known then maximum occupancy of guests used; Blue text= taken from SEC reports; [7, 26-59]

† indicates that numbers are approximate; when number of guests was not known, the maximum occupancy of guests was used

‡ indicates that numbers were taken from SEC reports.

Supplemental Table S3: Ships of less than 19 cases of COVID-19 from January 2020 to October 2020

Ship	Date Embarkation	Date Passenger Disembark-ation	Date Outbreak Identified	Location of Ship when Outbreak Identified	Number of Passengers at time of Outbreak	Number of Guests	Number of Crew	Number COVID-19 PCR Tests Performed	Total Known Cases	Known Guest Cases	Known Crew Cases	Total Known Deaths	Known Guest Deaths	Known Crew Deaths	Attack rate	Mortality rate	Notes
**Oasis of the Seas**	3/8/20	3/15/20	3/28/20	Great Harbor Cays, Bahamas	1,650	0	1,650	-	17	0	17	3	0	3	1.03	17.65	11 people medevacked
**MSC Opera**	2/17/20	2/28/20	3/4/20	Athens, Greece	2,785†	2,055‡	730‡	-	2	2	0	0	0	0	0.07	0.00	
**MSC Opera**	2/28/20	3/10/20	3/15/20	Genoa, Italy	2,785†	2,055‡	730‡	-	10	10	0	0	0	0	0.36	0.00	
**MSC Opera**	-	3/10/20	3/15/20	Genoa, Italy	200†	0	200†	-	16	0	16	1	0	1	8.00	6.25	
**MSC Bellissima**	2/21/20	2/28/20	-	Dubai, UAE	6,786†	5,386‡	1,400‡	-	2	2	0	0	0	0	0.03	0.00	
**MSC Bellissima**	2/29/20	3/7/20	-	Dubai, UAE	6,786†	5,386‡	1,400‡	-	5	5	0	0	0	0	0.07	0.00	
**MSC Bellissima**	3/7/20	3/14/20	3/19/20	Dubai, UAE	6,786†	5,386‡	1,400	-	15	13	2	0	0	0	0.22	0.00	
**Zaandam**	3/7/20	variable	3/27/20	(At Sea) from Buenos Aires to San Antonio,	1,829	1,243	586	-	15	7	8	9	7	2	0.82	60.00	Denied entry in Chile, unknown # of passengers transferred to Rotterdam, crew remained on board
**Coral Princess**	3/5/20	4/4/20	3/31/20	Barbados	1,898	1,020	878	-	13	8	5	6	5	1	0.68	46.15	
**MSC Preziosa**	>14 days no passengers	-	4/30/20	Ocean Cay, Bahamas	1,300†	0	1,300‡	-	13	0	13	0	0	0	1.00	0.00	data from audio of captain's announcement
**Marella Discovery 2**	>14 days no passengers	3/7/20	5/20/20†	-	735†	0	735‡	-	12	0	12	0	0	0	1.63	0.00	
**World Dream**	2/2/20	2/10/20	2/5/20	Hong Kong	3,691	1,871	1,820	1,800	12	12	0	0	0	0	0.33	0.00	
**Mein Schiff 3**	>14 days no passengers	3/23/20	5/1/20	Cuxhaven, Lower Saxony, Germany	2,899	0	2,899	15	10	0	10	0	0	0	0.34	0.00	usually max of staff is 1000 but it was higher as there were crew transferred from SCHIff4 and Schiff 6 for repatriation, not able to have 1 person per cabin. Riot on 5/2 due to conditions/lack of pay
**Enchantment of the Seas**	>14 days no passengers	-	6/14/20	Trinidad	309	0	309	-	9	0	9	0	0	0	2.91	0.00	some crew remained at sear for mor than three and a half months

**Black Watch**	>14 days no passengers	-	4/13/20	Rosyth, Scotland	330†	0	330‡	14	8	0	8	0	0	0	2.42	0.00	"Skeleton crew" on board so likely less than 330
**Norwegian Pride of America**	2/29/20	3/14/20	-	Honolulu, Hawaii, USA	3,113†	2,186‡	927‡	-	1	0	0	0	0	0	0.03	0.00	
**Norwegian Pride of America**	>14 days no passengers	3/14/20	4/8/20	Honolulu Hawaii, USA	550	0	50	-	7	0	7	0	0	0	1.27	0.00	
**MSC Fantasia**	3/13/20	3/23/20	3/24/20	Lisbon, Portugal	2,624†	1,299	1,325‡	39	3	3	0	0	0	0	0.11	0.00	Portuguese nationals first to disembark on 3/23, other nationalities remained on board until Portuguese nationals tested positive then began rapid disembarkment 3/24/20, 39 Portuguese nationals tested, British Australians, Brazilians left untested
**MSC Fantasia**	>14 days no passengers	3/22/20	4/11/20†	-	1,325†	0	1,325‡	-	7	0	7	0	0	0	0.53	0.00	crew recoding
**Marella Explorer 2**	3/15/20	3/26/20	3/25/20	Port Bridgetown, Barbados	980	200†	780‡	-	6	2	4	1	1	0	0.61	16.67	based on symptoms there were at least 19 passenger and 4 crew cases, unknown number repatriated to UK with flu like symptoms, 'many' staff with flu like symptoms
**Silver Explorer**	3/4/20	variable	3/14/20	Castro, Chile	231	111	120	-	6	6	0	0	0	0	2.60	0.00	
**Braemar**	2/27/20	3/17/20	3/9/20	St Maarten	1,512	1,128	384	-	5	1	4	0	0	0	0.33	0.00	The ship sat in the water for days without a solution with 682 passengers and 381 crew members. Had 28 guest and 27 crew on board with flu like symptoms that were not tested
**Costa Magica**	3/6/20	3/19/20	3/19/20†	Pointe-a-Pitre, Guadeloupe	3,269	2,309	960	-	5	4	1	0	0	0	0.15	0.00	after passengers disembarked, crew remained on board, 3/24/20 6 crew had flu like symptoms and were medevacked to Miami hospitals, COVID-19 results not found
**Fridtof Nansen**	3/7/20	3/17/20	3/20/20†	Portsmouth, England	488	388	100	-	4	4	0	1	1	0	0.82	25.00	
**Mariner of the Seas**	>14 days no passengers	-	4/1/20†	-	1,200†	0	1,200‡	-	4	0	4	0	0	0	0.33	0.00	5/10/20 Crew member found dead, unclear cause

**Norwegian Bliss**	3/1/20	3/8/20	3/17/20	New York City	6,930†	5,200‡	1,730‡	-	1	1	0	0	0	0	0.01	0.00	
**Norwegian Bliss**	3/1/20	3/8/20	3/17/20	New York City	6,930†	5,200‡	1,730‡	-	2	0	2	0	0	0	0.03	0.00	
**Norwegian Bliss**	>14 days no passengers	3/15/20	4/21/20†	FL, USA	1,730†	0	1,730‡	-	4	0	4	0	0	0	0.23	0.00	at least 100 ppl showed symptoms, 18 had 'pneumonias' and 4 were sent to Miami hospitals
**Costa Deliziosa**	>14 days no passengers	8/2/20	-	Rome, Italy	934†	0‡	934‡	-	4	0	4	0	0	0	0.43	0.00	
**MSC Grandiosa**	>14 days no passengers	3/19/20	4/6/2020†	Rome Italy	200	0	200	-	3	0	3	0	0	0	1.50	0.00	3 cases were all hospitalized
**Norwegian Breakaway**	2/29/20	3/7/20	3/18/20	Port Canaveral , FL	6,678†	5,088‡	1,590‡	-	1	1	0	0	0	0	0.01	0.00	
**Norwegian Breakaway**	3/7/20	3/14/20	-	Port Canaveral , FL	6,678†	5,088‡	1,590‡	-	3	3	0	0	0	0	0.04	0.00	numerous other passengers showed flu like symptoms but not tested, 2 cases from Westmorland County Pennsylvania thought from Norwegian Breakaway
**Celebrity Infinity**	3/9/20	3/14/20	3/23/20	Port Tamp, FL, USA	3,449†	2,449‡	1,000‡	-	1	1	0	0	0	0	0.03	0.00	crew left on 5/21/20, positive on 5/23/20
**Celebrity Infinity**	>14 days no passengers	3/14/20	3/29/20	Miami, FL, USA	1,000†	0	1,000‡	-	2	0	2	1	0	1	0.20	50.00	
**Liberty of the Seas**	>14 days no passengers	-	3/30/20	Galveston , TX, USA	1,235	0	1,235	-	2	0	2	0	0	0	0.16	0.00	11 not confirmed crew were isolated with high temperatures
**MS Queen Victoria**	>14 days no passengers	early march	4/17/20†	Southampton, England	1,253†	0	1,253‡	-	2	0	2	0	0	0	0.16	0.00	source from leaked audio
**MSC Meraviglia**	3/1/20	3/8/20	3/12/20	Miami, FL, USA	6,786	5,386‡	1,400‡	-	1	1	0	0	0	0	0.01	0.00	
**MSC Meraviglia**	3/8/20	3/15/20	-	Miami, FL, USA	5,277	3,877	1,400	-	2	2	0	0	0	0	0.04	0.00	Passengers not screened on disembarkation
**MSC Orchestra**	3/13/20	3/16/20	3/27/20†	Mozambique (test in New castle)	4,487†	3,500	987‡	-	2	1	0	0	0	0	0.04	0.00	earliest symptoms 3/13-3/16, unclear test date
**Seven Seas Navigator**	>14 days no passengers	-	5/21/20	Barcellona, Spain	450†	0	450†	2	2	0	2	0	0	0	0.44	0.00	
**Silver Shadow**	3/7/20	3/22/20	3/12/20	Recife, Brazil	609	318	291	3	2	2	0	1	1	0	0.33	50.00	Different articles give total and # guests and crew, hence the discrepancy. Passengers spent 10 days the on cruise ship awaiting disembarkation, crew remained on board

**Sun Princess**	1/29/20	3/19/20	3/26/20	Fremantle, Australia	2,900	2,000	900‡	-	1	1	0	0	0	0	0.03	0.00	Turned away from Madagascar and met with protests on Reunion island; Some passengers disembarked on Kangaroo Island on March 16th, 2020, and the rest disembarked in Sydney on March 19th, 2020
**Carnival Imagination**	3/5/20	-	-	-	3,514†	2,594‡	920‡	-	1	0	0	0	0	0	0.03	0.00	
**Carnival Imagination**	3/8/20	-	-	-	3,514†	2,594‡	920‡	-	1	1	0	0	0	0	0.03	0.00	
**Carnival Imagination**	3/12/20	-	-	-	3,514†	2,594‡	920‡	-	1	1	0	0	0	0	0.03	0.00	
**Carnival Valor**	2/29/20	3/5/20	-	New Orleans, USA	4,860†	3,710‡	1,150‡	-	1	1	0	0	0	0	0.02	0.00	
**Carnival Valor**	3/5/20	3/9/20	-	New Orleans, USA	4,860†	3,710‡	1,150‡	-	1	0	0	0	0	0	0.02	0.00	
**Carnival Valor**	3/9/20	3/14/20	-	New Orleans, USA	4,860†	3,710‡	1,150‡	-	1	0	0	0	0	0	0.02	0.00	
**Carnival Vista**	2/15/20	2/22/20	-	Galveston, TX, USA	5,404†	3,954‡	1,450‡	-	1	0	0	0	0	0	0.02	0.00	
**Carnival Vista**	2/29/20	3/7/20	-	Galveston, TX, USA	5,404†	3,954‡	1,450‡	-	1	1	0	0	0	0	0.02	0.00	
**Celebrity Reflection**	3/13/20	3/23/20	-	Fort Lauderdale, USA	4,500†	3,000‡	1,500‡	-	1	0	0	0	0	0	0.02	0.00	
**Celebrity Silhouette**	2/23/20	3/6/20	3/10/20	Ft. Lauderdale, FL, USA	4,500†	3,000‡	1,500‡	-	1	1	0	0	0	0	0.02	0.00	
**Celebrity Summit**	2/29/20	3/7/20	-	San Juan. Puerto Rico, USA	3,449†	2,449‡	1,000‡	-	1	0	0	0	0	0	0.03	0.00	
**Crown Princess**	3/6/20	3/16/20†	-	Ft. Lauderdale, FL, USA	5,000†	3,800‡	1,200‡	-	1	0	1	0	0	0	0.02	0.00	
**Crown Princess**	>14 days no passengers	3/16/20†	-	Ft. Lauderdale, FL, USA	1,200†	0	1,200‡	-	1	0	1	1	0	1	0.08	100.00	
**Explorer of the Seas**	3/8/20	3/15/20	-	Miami, FL, USA	4,294†	3,114†	1,180†	-	1	0	0	0	0	0	0.02	0.00	
**Harmony of the Seas**	>14 days no passengers	-	-	-	2,100†	0	2,100‡	-	1	0	1	0	0	0	0.05	0.00	
**Majesty of the Seas**	2/29/20	3/7/20	3/14/20	Gulfport, MS, USA	3,588	2,676	912	-	1	0	0	0	0	0	0.03	0.00	

**Pullmantur Monarch**	>14 days no passengers	-	4/17/20†	Panama City, Panama	803†	0	803†	-	1	0	1	1	0	1	0.12	100.00	
**MS Marina**	>14 days no passengers	variable, before 3/23/20	-	Miami, FL, USA	800†	0	800‡	-	1	0	1	0	0	0	0.13	0.00	
**MSC Armonia**	3/2/20	3/9/20	-	Miami, FL, USA	2,825†	2,065‡	760‡	-	1	1	0	0	0	0	0.04	0.00	
**Norwegian Encore**	3/8/20	3/15/20	3/22/20	Miami, FL, USA	1,000†	0	1,000†	0	1	0	1	0	0	0	0.10	0.00	"Dozens" were sick but not tested
**Norwegian Gem**	>14 days no passengers	-	4/13/20	Great Stirrup Cay, Bahamas	1,070†	0	1,070‡	-	1	0	1	0	0	0	0.09	0.00	3 deaths, including MD in cabin, not allowed to get off to get tests
**Oceania Riviera**	2/26/20	3/11/20	-	Miami, FL, USA	2,100	1300	800	-	1	0	0	0	0	0	0.05	0.00	
**MS Paul Gaugin**	7/30/20	8/2/20	8/1/20	Papette, Tahiti	340	147	192	-	1	1	0	0	0	0	0.29	0.00	
**Queen Mary 2**	-	3/14/20	3/31/20	Durban, South Africa	1,226	264	1,215	27	1	0	1	0	0	0	0.08	0.00	
**Rhapsody of the Seas**	3/7/20	3/14/20	-	Tampa, FL, USA	3,181†	2,416‡	765‡	-	1	0	0	0	0	0	0.03	0.00	
**Scarlet Lady**	Not yet had first voyage	-	3/22/20	Miami, FL, USA	1,160†	0	1,160‡	-	1	0	1	0	0	0	0.09	0.00	had not yet had maiden voyage
**Sea Dream**	7/21/20	7/28/20†	8/4/20	Oslo, Norway	195†	110†	85†		1	1	0	0	0	0	0.00	0.00	
**Sky Princess**	>14 days no passengers	"beginning of March"	-	Port Canaveral , FL, USA	1,300	0	1,300	-	1	0	1	0	0	0	0.08	0.00	
**Symphony of the Seas**	3/7/20	3/14/20	3/30/20	Everglade s, FL, USA	2,390†	0	2,390‡	-	1	0	1	1	0	1	0.04	100.00	up to 31 additional cases as per anonymous report, (including 2 nurses)
**Westerham**	2/1/20	2/13/20	2/15/20	Sihanoukv ille, Cambodia	1,528	781	747	-	1	0	0	0	0	0	0.07	0.00	

Supplemental Table S3: Cruises with less than 20 cases; [12 Red text = Approximate, when number of guests not known then maximum occupancy of guests used; Blue text= taken from SEC reports;, 28, 41, 59, 60-140]

† indicates that numbers are approximate; when number of guests was not known, the maximum occupancy of guests was used

‡indicates that numbers were taken from SEC reports.

Additional References

1. Farrell P, McDonald A. Thousands of cruise ship passengers told to self-isolate due to coronavirus days after disembarking. 2020 3/23/3030.
2. Uki Goñi. Cruise ship stranded off Uruguay says 60% onboard have Covid-19. 4/7/2020.
3. Ing AJ, Cocks C, Green JP. COVID-19: in the footsteps of Ernest Shackleton. Thorax. 2020;75(8):693-4.
4. Harris A. 'They absolutely did not care': Crew members sue Celebrity Cruises over virus response. The Miami Herald. 2020 4/14/2020.
5. Crew Center. Pullmantur Horizon Crew Reports COVID-19 Positive Cases Onboard. Crew Center. 2020 04/04/2020.
6. The Maritime Executive. Fifty-Seven New COVID-19 Cases on Costa Atlantica. The Maritime Executive. 4/26/2020.
7. Mina Kaji, Amanda Maile, Benitez G. Passengers sue Celebrity over 'heightened risk' of COVID-19 exposure. abc News. 2020 5/18/2020.
8. Hope H. 1 passenger, 3 crew members on Celebrity Eclipse cruise ship test positive for COVID-19. CBS8. 3/31/2020.
9. Ship Technology. Ruby Princess, Cruise Liner, Bermuda [Available from: https://www.ship-technology.com/projects/ruby_princess/.
10. Travel Online. Travel Online [Available from: https://www.travelonline.com/.
11. COSTA ATLANTICA PROFILE PAGE Beyond Ships [Available from: https://www.beyondships.com/CostaAtlarntica-Profile.html.
12. Cruise Mapper. [Available from: https://www.cruisemapper.com/.
13. Royal Caribbean Cruises. Ovation of the Seas Fact Sheet [Available from: https://www.royalcaribbeanpresscenter.com/fact-sheet/28/ovation-of-the-seas/.
14. Weekly T. [Available from: https://www.travelweekly.com/.
15. OZ Cruising. [Available from: https://www.ozcruising.com.au/.
16. Cruise Critic.
17. Royal Caribbean Cruises. Royal Caribbean Cruises 2020 [Available from: https://www.royalcaribbean.com/cruise-ships.
18. Black Watch Itinerary 2020 [Available from: https://crew-center.com/black-watch-itinerary.
19. Norwegian Cruise Line [Available from: https://www.ncl.com/#.
20. Line NC. SEC Filings 2020 [Available from: http://www.nclhltdinvestor.com/financial-information/sec-filings.
21. Costa Cruises. Our Fleet 2020 [Available from: https://www.costacruises.com/fleet.html.
22. Royal Caribbean's Explorer of the Seas - March 8, 2020 Sunday @ 10:00 pm [Available from: http://dslegacy.com/shows/2020-03-08/.
23. Lipsey S. On board the world's biggest cruise ship. CNN. 2020 5/20/2016.
24. Pullmantur Cruises [Available from: https://www.pullmanturcruises.de/.
25. RMS Queen Mary 2 Ship.
26. Marc Leras. Cruise ship bound for Italy had 36 coronavirus cases during Marseille stop. Reuters. 2020 3/20/2020.
27. iCruise [Available from: https://www.icruise.com/.
28. Covid-19: Costa Luminosa cruise ship docks in Marseille, France. Ship Technology. 2020 3/20/2020.
29. Austin Carr, Chris Palmeri. Carnival Executives Knew They Had a Virus Problem, But Kept the Party Going. Bloomberg. 2020.

30. Taylor Dolven. Dozens reported sick as Costa cruise ship nears France with 233 Americans on board. Miami Herald. 2020 3/18/2020.
31. Government Statement on Passing of COVID-19 Patient [press release]. 3/14/2020 2020.
32. AARON FERNANDES, CAMILLE BIANCHI. WA cruise ship standoff continues as passengers test positive for COVID-19. SBS News. 2020 3/26/2020.
33. Sophia Ankel. One of the last cruises at sea now has just 8 passengers left after 4 died of the coronavirus and hundreds abandoned ship. Here's how a 140-day world tour turned to disaster. Business Insiders. 2020 4/24/2020.
34. Francesca Street. After months at sea, the final cruise ship carrying passengers makes it home. CNN. 2020 6/8/2020.
35. Fernanda Balbino. Number of Crew Members Infected by Coronavirus in MSC Seaview Rises to 86. A Tribuna. 2020 5/5/2020.
36. ATribuna.com.br. With 10 Cases fof COVID-19, MSC Seaview Quarantines in the Port of Santos. A Tribuna. 2020 4/30/2020.
37. Ginger Jeffries. Celebrity Eclipse cruise ship passenger dies from COVID-19. KUSI News. 2020 4/9/2020.
38. Ginger Jeffries. County confirms passenger on Celebrity Eclipse Cruise Ship tests positive for COVID-19. KUSI News. 2020 3/30/2020.
39. HLN. Gestorven na droomcruise die nooit had mogen uitvaren. HLN. 2020.
40. Taylor Dolven. Two Costa cruise ships plan to dock at PortMiami Thursday with 30 sick on board. Miami Herald. 2020 3/25/2020.
41. Taylor Dolven. Costa Favolosa cruise ship crew member dies in Miami after getting COVID-19 on board. Miami Herald. 2020 4/6/2020.
42. Sarah McPhee. Five cruises from NSW infected with virus. Daily Mercury. 2020 3/21/2020.
43. Coronavirus claims life of Toowoomba man infected on cruise ship who was allowed to travel home. ABC News. 2020 3/25/2020.
44. Hutcheon H. More cruise ships bring coronavirus-infected passengers to Sydney. Seatrade Cruise News. 2020 3/21/2020.
45. G1 Santos. Navio atracado em Santos tem 20 casos confirmados e seis suspeitos de coronavírus. G1. 2020 4/13/2020.
46. John Wayne Fergusen. Carnival tells Freedom passengers to isolate after crew member tests positive. The Daily News. 2020 3/26/2020.
47. Stanton S. After trip to Antarctica, Sacramento attorney says he and his wife have coronavirus. Sacramento Bee. 2020.
48. Ann Kalosh. At least 36 Hurtigruten crew and one passenger have COVID-19. 2020 8/1/2020.
49. Solsvik T. At least 40 infected with COVID-19 on Norway cruises amid scramble to trace passengers. Reuters. 2020, 8/2/20.
50. Taylor Dolven, Charles J. COVID-19 outbreak on Royal Caribbean ship delays homecoming for Trinidadian crew. Miami Herald. 2020 6/30/2020.
51. 29 Vincentian Seafarers aboard Vision of the Seas Test Positive for Covid-19 Exposure. 2020 5/27/20.
52. Eight new COVID-19 cases; total now 564. Loop News. 5/26/2020.
53. Garwin Davis. Ship Workers Arrive At Falmouth Pier Jamacia Information Service2020 [updated 5/20/2020. Available from: https://jis.gov.jm/ship-workers-arrive-at-falmouth-pier/.
54. 25 MSC Splendida crew diagnosed with COVID-19. ABC News. 2020 4/21/2020.
55. MSC Splendida Emergency Medical Evacuation of Crew Member. Crew Center. 2020 3/23/2020.
56. Hines M. COVID-19 chaos: MSC Fantasia passengers 'kept in the dark' until day of disembarkation. USA Today. 2020 4/14/2020.

57. Coronavirus: Fears on cruise ship docked at Italian port after case confirmed. The Local. 2020 3/25/2020.
58. COVID-19 Update on Ruby Princess and Celebrity Solstice cruises [press release]. New Zealand Ministry of Health, 3/20/2020 2020.
59. Blaskey S, Nehamas N, Dolven T. COVIDCRUISES_MIAMI_HERALD_DATA. In: Herald M, editor. 2020.
60. Gorton S. Coronavirus COVID-19 case link to Sun Princess cruise ship that visited Kangaroo Island. The Island. 2020 3/28/2020.
61. DEVOUN CETOUTE, TAYLOR DOLVEN. 14 crew have COVID-19, Royal Caribbean captain says, after passengers got off in Miami. Mia,mi Herald. 3/29/2020.
62. Walker J. Third Crew Member from the Oasis of the Seas Dies Due to COVID-19. Cruise Law News. 2020.
63. NEAL DJ, DOLVEN T. A third crew member from Royal Caribbean's Oasis of the Seas dies from COVID-19. Bradenton Herald. 2020 5/4/2020.
64. Braine T. Two Royal Caribbean Oasis of the Seas crew members die of COVID-19 in two days. Daily News. 2020 4/21/2020.
65. MSC Opera Status: 200 crew onboard, 7 positive for COVID-19 at home, 13 in quarantine on the ship. Crew Center. 2020 3/21/20.
66. Long KK. Circus performer from Seattle trapped aboard Persian Gulf cruise ship due to coronavirus lockdown. Seattle Times. 2020 4/10/2020.
67. TelanganaToday. Two more persons test positive for COVID-19 in Telangana. Telangana Today. 2020 3/21/2020.
68. Dolven T, Gross SJ, Harris A. Zaandam cruise ship with COVID-19 on board docks in Florida after 12 days at sea. Anchorage Daily News. 2020 4/2/2020.
69. Brackett R. Governor Says State Will Accept Florida Residents from Cruise Ship Stricken with Coronavirus. The Weather Channel. 2020 4/1/2020.
70. Harris S. 'Get us the hell off': Nearly 250 Canadians aboard ship with passengers, crew exhibiting flu-like symptoms. CBC. 2020 3/23/2020.
71. Harris A. Zaandam cruise ship gets permission to cross Panama Canal and head to Port Everglades. Miami Herald. 2020 3/28/2020.
72. Harris A. Four passengers died on Zaandam cruise ship; 148 aboard sick with flu-like symptoms. Miami Herald. 2020 3/27/2020.
73. Coral Princess Updates [press release]. 3/9/2020 2020.
74. Harris A, Kirpalani R. Coast Guard blocks Coral Princess from docking in Port Everglades until it has a plan. Miami Herald. 2020 4/3/2020.
75. Vidal G. Austin woman tests positive for COVID-19 over a week after being quarantined on cruise. CBS Austin. 2020 4/16/2020.
76. Dolven T. No information. No way off. 100,000 crew members remain in cruise ship limbo for months. Miami Herald. 2020 5/17/2020.
77. MSC Preziosa: 2 crew members test positive for Covid-19. Crew Center. 2020 5/1/2020.
78. Jamaicans from Marella Discovery 2 who tested positive for COVID-19 blame gov't for them contracting virus. Radio Jamacia News. 2020 5/21/2020.
79. Mumbai Mirror. 750 seafarers stuck on ships can now disembark. Mumbai Mirror. 220 4/23/2020.
80. Coronavirus: Thousands on cruise ship allowed to disembark after tests. BBC. 2020 2/10/2020.
81. Coronavirus: World Dream cruise ship quarantined in Hong Kong. Ship Technology. 2020 2/6/2020.

82. The Associated Press. Passengers leave Hong Kong cruise ship after coronavirus quarantine lifted. CBC. 2020 2/9/2020.
83. Denyer S, O'Grady S. Passengers quarantined on cruise ship are desperate to escape coronavirus that infected 64 fellow travelers. Wachington Post. 2020 2/7/2020.
84. After Intense Bargaining TUI Cruises Begin Seafarers' Repatriation, 9 Seafarers Infected. Maritime POst. 2020 5/20/2020.
85. Dolven T, Charles J. COVID-19 outbreak on Royal Caribbean ship delays homecoming for Trinidadian crew. Miami Herald. 2020 6/30/2020.
86. Nanton S. 5 more COVID-19 cases push the figure up to 123. These 5 and 1 earlier today all came from the 308 who returned on the Enchantment of the Seas cruise ship on Friday. In: @samnanton, editor. 2020.
87. Coronavirus: Eight crew of Black Watch cruise liner test positive. BBC. 2020 4/13/2020.
88. Peterson B, Matousek M, Cain Á. Cruise lines told ship workers to carry on as normal as the coronavirus spread. Now, many crew members are infected or unemployed. Business Insider. 2020 4/7/2020.
89. HNN Staff. 7 crew members on cruise ship at Honolulu Harbor test positive for COVID-19. Hawaii News Now. 2020 4/8/2020.
90. macronews.com. COVID-19 chaos: MSC Fantasia passengers 'kept in the dark' until day of disembarkation. News Break. 2020 3/24/2020.
91. Dolven T. 'I don't feel safe at all.' As cruise ships sail on, crews fear COVID-19 infection. Miami Herald. 2020 3/24/2020.
92. The Associated Press. Mexico allows 46 British passengers to leave cruise ship. ABC News. 2020 4/1/2020.
93. Yucatan reports two COVID-19 related deaths. Yucatan Times. 2020 4/5/2020.
94. IGLU CRUISE [Available from: https://www.iglucruise.com/.
95. Woodyard C, Hines M. Celebrity, Costa Cruises work to bring back stranded ships, get passengers home. USA Today. 2020 3/18/2020.
96. Zdanowicz C. Multiple cruise ships are left stranded as coronavirus cases increase. CNN. 2020 3/17/2020.
97. Robles F. Passengers Fell Ill With Coronavirus. And the Ship Sailed On. New York Times. 2020 3/19/2020.
98. Radia K, Carrega C. Coronavirus-infected cruise ship stranded at sea for weeks to dock in Cuba. ABC News. 2020 3/17/2020.
99. Street F. Virus-hit cruise ship MS Braemar docks in Cuba after Caribbean odyssey. CNN. 2020 3/18/2020.
100. AFP Staff. French passengers sue Costa Cruises over virus ship ordeal. CTV News. 2020 8/9/2020.
101. Hussein SF. Virus-Affected Costa Magica Cruise Ship In Caribbean Carrying 38 Russians - Embassy. Urdu Point. 2020 3/13/2020.
102. 'Guest' died after Hurtigruten cruise. NEWSinENGLISHno. 2020 8/26/2020.
103. Ocasio BP, Dolven T. Desperate cruise employees say they're losing hope amid reports of overboard deaths. Miami Herald. 2020 5/13/2020.
104. Grundland M. 2-year-old passenger on Norwegian Bliss tested positive for coronavirus. Silive. 2020 3/17/2020.
105. Walker J. Positive COVID Crew Cases Rise to Nine on Costa Favolosa and Costa Deliziosa. Cruise Law News. 2020 8/9/2020.
106. Centrale R. Civitavecchia Porto – Tre marittimi di Costa positivi al test Covid-19 in pochi giorni. Etruria News. 2020 8/2/2020.

107. Delahaye J. Coronavirus: MSC Cruises cancels cruises across eight ships - see the full list. Mirror. 2020 3/12/2020.
108. Sostek A. A cough, a cruise ship and a Westmoreland County case of coronavirus. Pittsburgh Post-Gazette. 2020 3/24/2020.
109. Kosik A. Crew member aboard Celebrity Infinity raises concerns over whether cruise ship unnecessarily put crew at risk. CNN. 2020 4/6/2020.
110. Powell N. Two Royal Caribbean cruise crew members test positive for coronavirus in Galveston. Houston Chronicle. 2020 4/1/2020.
111. Cain A. Leaked audio reveals that crew members on Cunard Line's MS Queen Victoria cruise ship off the coast of England have been asked to quarantine in their rooms because of COVID-19 cases on board. 2020 4/18/2020.
112. Coronavirus: Cunard ends its three world cruises. BBC. 2020 3/16/2020.
113. Dolven T. MSC cruise passengers posed ‘medium risk.’ CDC cleared them to disembark in Miami anyway. Miami Herald. 2020 3/18/2020.
114. Naicker E. MSC passenger’s Covid-19 results released by NICD. Northern Natal News. 2020 3/27/2020.
115. Roodepoort. TRAVELLING ON THE MSC ORCHESTRA AFTER PASSENGERS TEST POSITIVE FOR COVID-19. Oudtshoorn Courant. 2020.
116. Cai B. Un crucero con 450 ocupantes, en cuarentena en el puerto de Barcelona por dos posibles positivos. El Pais. 2020 5/21/2020.
117. Hierro J. Tripulantes del crucero atrapado por el Covid: «Estamos bien, pero queremos volver a casa. ABC. 2020 5/23/2020.
118. Woodyard C, Hines M. 103 passengers from Silver Shadow cruise ship flown to US after coronavirus ordeal. USA Today. 3/24/2020.
119. Humphreys A. Canadian man dies from COVID-19 in Brazil hospital after being taken off cruise ship. National Post. 2020 3/27/2020.
120. G1 PE. Idoso passa mal em cruzeiro, e navio com centenas de pessoas atracado no Recife é isolado por suspeita de coronavírus. G! 2020 3/12/2020.
121. Henry D. Coronavirus: Violent protests meet cruise ship full of Kiwis, Australians at Réunion Island. New Zealand Herald. 2020 3/1/2020.
122. Hines M. Thousands may have been exposed to coronavirus on Princess cruise ship; 62 passengers confined. USA Today. 2020 3/4/2020.
123. NBC4 Staff. Coronavirus in Ohio: First COVID-19 case confirmed in Columbus. NBC4. 2020 3/14/2020.
124. 5th case of COVID-19 in Waterloo region went on plane with symptoms, public health says. CBC. 2020 3/17/2020.
125. WLOX Staff. Royal Caribbean cruise ship arrive in Gulfport to disembark crew members. WLOX. 2020 3/24/2020.
126. In Memoriam: Cruise Ship Crew Members Lost in COVID-19 Pandemic. Crew Center. 2020 4/7/2020.
127. COVID-19 Update: 17 More Recoveries, 4 New Imported Cases Recorded [press release]. 6/5/2020 2020.
128. Three Puerto Rican Crew Members Are Repatriated Aboard The MS Marina. Maritime Herald. 2020 6/23/2020.
129. Jordan AE. More Cruise Ships Reach Port. The Maritime Executive. 2020 3/23/2020.
130. Dolven T. Crew are stuck on Miami cruise ships with COVID-19 spreading. Some aren’t being paid. Miami Herald. 2020 3/29/2020.

131. Peterson B. A doctor has died on the Norwegian Gem, two weeks after crew members saw another body loaded into an ambulance. Buisness Insider. 2020 5/1/2020.
132. Rogers S. A cruise ship that had at least one passenger test positive for COVID-19 may be coming to Eastport. NBC News Center Maine. 2020 5/13/2020.
133. Valandina M, Kalosh A. Apart from one COVID-19 case on aborted Paul Gauguin cruise, everyone else tests negative. Seatrade Cruise News. 2020 8/3/2020.
134. Queen Mary 2 stocks up, leaves Durban after dropping off 6 South Africans. News 24. 2020 4/2/2020.
135. Centers for Disease Control and Prevention. CDC's role in helping cruise ship travelers during the COVID-19 pandemic 2020 [updated 11/23/2020. Available from: https://www.cdc.gov/coronavirus/2019-ncov/travelers/cruise-ship/what-cdc-is-doing.html.
136. Cetoute D, Dolven T. Virgin Voyages crew member dies on ship, Coast Guard says. Another in cruise industry deaths. Miami Herald. 2020 3/24/2020.
137. Asymptomatic SeaDream Guest Tests Positive for COVID-19 After Arriving in Denmark [press release]. 8/4/2020 2020.
138. Edlund A, Poulisse A. Coronavirus: Hundreds of crew members stuck on cruise ship for 40 days off Florida coast. Kiro 7. 2020 4/25/2020.
139. Walker J. Four Additional Royal Caribbean Crew Members with COVID-19 Evacuated from Oasis of the Seas. Cruise Law News. 2020 4/8/2020.
140. Thakkar E. Symphony of the Seas Crew Member Dies from COVID-19. Cruise Hive. 2020 4/15/2020.
